# Hydrophobized MFC as Reinforcing Additive in Industrial Silica/SBR Tire Tread Compound

**DOI:** 10.3390/polym15193937

**Published:** 2023-09-29

**Authors:** Ming Liu, Iikpoemugh Elo Imiete, Mariapaola Staropoli, Pascal Steiner, Benoît Duez, Damien Lenoble, Emmanuel Scolan, Jean-Sébastien Thomann

**Affiliations:** 1 Material Research and Technology Department (MRT), Luxembourg Institute of Science and Technology (LIST), 41 Rue du Brill, L-4422 Belvaux, Luxembourg; mingliu_0603@yahoo.com (M.L.);; 2 Goodyear Innovation Center Luxembourg (GIC*L), Avenue Gordon Smith, L-7750 Colmar-Berg, Luxembourg

**Keywords:** micro fibrillated cellulose, surface functionalization, silica reinforcement, nanocomposite, elastomer

## Abstract

Silica is used as reinforcing filler in the tire industry. Owing to the intensive process of silica production and its high density, substitution with lightweight bio-based micro fibrillated cellulose (MFC) is expected to provide lightweight, sustainable, and highly reinforced tire composite. MFC was modified with oleoyl chloride, and the degree of substitution (DS) was maintained between 0.2 and 0.9. Subsequently, the morphology and crystallinity of the modified MFC were studied and found to be significantly dependent on the DS. The advantages associated with the use of the modified MFC in synergy with silica for the reinforcement of styrene butadiene rubber (SBR) nanocomposite was investigated in comparison with silica/SBR compound. The structural changes occasioned by the DS values influenced the processability, curing kinetics, modulus-rolling resistance tradeoff, and tensile properties of the resultant rubber compounds. We found that the compound made with modified MFC at a DS of 0.67 (MFC16) resulted to the highest reinforcement, with a 350% increase in storage modulus, 180% increase in Young`s modulus, and 15% increase in tensile strength compared to the referenced silica-filled compounds. Our studies show that MFC in combination with silica can be used to reinforce SBR compound for tire tread applications.

## 1. Introduction

There is a huge drive to replace petrochemical-based materials with renewable resources in various applications (e.g., packaging, glass-fiber-reinforced composites, tire compounds, etc.). For example, the tire industry utilizes vast amounts of non-renewable fillers for reinforcement, such as carbon black and silica. Carbon black is derived from fossil fuel, and silica is derived from minerals. Apart from their non-renewable nature, silica has an undesirably high density, and its production is both time and energyconsuming [1,2,3]. Cellulose and cellulose derivatives such as micro fibrillated cellulose (MFC) constitute abundant, inexpensive, lightweight, and renewable fillers [4,5] compared to carbon black and silica. The potential use of cellulosic derivatives as a reinforcing filler has been demonstrated in several publications [6,7,8]. The drawback of cellulose is its hydrophilic character, which results to poor dispersion and weak interfacial adhesion when incorporated in hydrophobic polymer for composite fabrication. Therefore, modification of cellulosic fibers is necessary to avoid these drawbacks for improved performance of cellulose-based composites.

The use of modified cellulose fibers in combination with the currently used fillers could be a robust approach targeted at incorporating MFC and gradually phasing out non-renewable fillers. This could be achieved by the addition or partial substitution of silica and carbon black with MFC, which may lead to a more sustainable design of innovative compounds useful for the tire industry. In this respect, our group has demonstrated recently that a combination of fillers within styrene butadiene rubber (SBR)-based nanocomposites results in novel mechanical reinforcement behavior [9]. Therefore, the use of bio-sourced micro fibrillated fibers such as MFCs in combination with silica in rubber could possibly enhance the synergy previously observed between silica and anisotropic structures.

Cellulose fibers and other cellulose derivatives have been combined with silica and carbon black in rubber matrices with very promising results [10,11,12,13]. These studies showed that the dispersion and mechanical properties would need to be improved when the matrix is highly hydrophobic, such as SBR. The high hydrophobicity of SBR would therefore require an efficiently modified MFC to fit as a reinforcing agent. The modification process of MFC often leads to loss of crucial fiber properties [14] which are needed for reinforcement.

A limited number of studies have been dedicated to filled rubber compounds prepared by melt mixing MFCs into elastomers. One study was reported in 2018 [15] using a melt-mixing process with chemically modified freeze-dried MFCs in SBR. The freeze-dried MFCs were esterified with palmitoyl chloride or 3,3′dithiopropionic acid chloride using a gas-phase protocol and subsequently incorporated into rubber elastomers using an internal mixer. This result demonstrated that hydrophobized MFCs could be successfully incorporated into rubber elastomers using current industrially applied melt-mixing processes. The properties of the resulting MFC composite were promising. However, the freeze-drying processing before and after the surface functionalization of MFCs cannot be easily upscaled for large-scale industrial production. Also, the storage modulus offers the possibility of improvement.

Water removal is required for MFC modification and is crucial, as several modification reactions are sensitive to moisture. Optimizing the process of water removal by avoiding freeze or oven drying is important to reduce cost and preserve the fibrillar morphology, which is desirable for high levels of reinforcement. Alternatively, there are reports of hydrophobic modification of cellulose derivatives that are not sensitive to water [16,17]. However, they are governed by reaction equilibria, the use of excess reactants, and low reaction yield, which negatively impact on reproducibility. These processes would be difficult to upscale, with reproducibility and cost as the main drawbacks. These studies, at best, present the potentials of MFCs and do not outline key aspects that can translate laboratory results into industrial compounds. One of the disconnects between laboratory experiments and industrial manufacturing is the effective replication of processing condition. We considered these drawbacks in our studies and went a step further to study the processability of the fabricated compounds, which is crucial for upscaling.

In this study, we developed a scalable process using modified MFCs to substitute different amounts of silica in MFC/Silica/SBR nanocomposites. The MFCs were modified with oleoyl chloride at various degrees of substitution (DS) to hydrophobize the MFC surface and facilitate dispersion in the SBR matrix. The same role can be assigned to TDAE oil, which facilitates the incorporation of filler into polymeric matrices. Therefore, we modulated the amount of processing oil in the recipe to compensate for a higher DS while maintaining a filler level needed to meet the percolation threshold. MFC modification started with solvent-exchanging MFCs slurry from water to DMAc using a rotary evaporator. Thereafter, the MFCs were functionalized and studied with FTIR, XRD, SEM, and TGA. Finally, the modified MFCs were melt-mixed with nanosilica and SBR in a Brabender internal mixer. The properties of the resulting composites were studied to understand the effect of the DS, TDAE oil, and filler amount on the mechanical properties and processability of the resultant elastomeric compounds.

## 2. Materials and Methods

### 2.1. Materials

Micro fibrillated cellulose (MFC) slurry (Exilva F01V) with solid content of about 12 wt.% was purchased from Borregaard, Sarpsborg, Norway. Dimethylacetamide (DMAc) and tetrahydrofuran (THF) were purchased from Carl Roth (Karlsruhe, Germany). Oleoyl chloride (˃89%) and anhydrous pyridine were purchased from Sigma-Aldrich (St. Louis, MO, USA), while polybutadiene (PB), styrene butadiene rubber (SBR), treated distillate aromatic extract (TDAE oil), zinc oxide, stearic acid, nano silica (200 nm), Bis(triethoxysilylpropyl)disulfide (TESPD), N-(1,3-dimethylbutyl)-N′-phenyl-p-phenylenediamine (6PPD), sulfur, 2-mercaptobenzothiazole (MBT), diphenyl guanidine (DPG), and N-cyclohexyl-2-benzothiazylsulfenamide (CBS) were provided by Goodyear Tire and Rubber Company, Luxembourg.

### 2.2. MFC Modification

**Figure 1:**
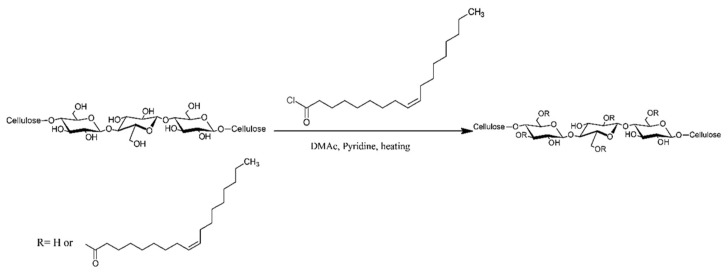
Schematic drawing for the esterification reaction between cellulose and oleoyl chloride.

The esterification of MFCs with oleoyl chloride was carried out in DMAc solution after solvent exchanging the MFC slurry from water to DMAc using a rotary evaporator. The reaction shown in Figure 1 is an addition/elimination reaction wherein the fatty acid derivative is grafted on the hydroxyl groups of MFCs, with the formation of pyridinium chloride as a byproduct. The process used about 15 g MFCs slurry dispersed in 150 g DMAc with a high shear homogenizer for 5 min to form a homogeneous MFC slurry. The MFC slurry was then concentrated using a rotary evaporator under a vacuum of 50 mbar and a temperature of 80 °C until 80–90 g water had been evaporated. Subsequently, an equal amount of DMAc (80–90 g) was poured into the concentrated MFC slurry, and the mixture was further homogenized with a homogenizer for 1 min at 15,000 rpm. The new MFC slurry was concentrated using a rotary evaporator at an elevated vacuum of 20 mbar at 90 °C until half of the initial mass had been lost. The final MFC in DMAc solution for esterification was obtained by further homogenization in DMAc with an adjusted concentration of 1% dry matter content of MFC. The wight of MFC was determined gravimetrically after drying the solution under 100 mbar at 105 °C for 24 h.

After the solvent exchange, the esterification of MFC proceeded by adding a certain amount of pyridine, MFC/DMAc slurry, and oleoyl chloride in a reaction chamber under a nitrogen atmosphere and stirred at 90 °C and 1000 rpm for 24 h using a Carousel Tornado overhead stirring system. The molar ratio of the oleoyl chloride/OH group of MFCs was between 0.3 and 0.9, and the molar ratio of oleoyl chloride/pyridine was 1.0. After esterification, the modified MFCs were washed three times with THF using a centrifuge. The degree of substitution (DS) of modified MFCs was determined based on the gained mass of the modified MFCs and the initial mass of MFCs before esterification [18]. By varying the molar ratio of the oleoyl chloride/OH group of MFCs, modified MFCs with low DS (LDS) of 0.24, medium DS1 (MDS1) of 0.49, medium DS2 (MDS2) of 0.67, and high DS (HDS) of 0.91 were obtained.

### 2.3. Composite Preparation

**Figure 2:**
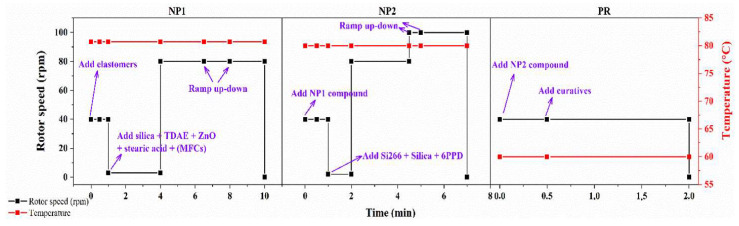
Applied compounding parameters, including rotor speed and temperature of the mixing chamber in the NP1, NP2, and PR stages.

The compounding of rubber composites was performed using a HAAKE PolyLab OS internal mixer (Thermo Scientific) with a mixing chamber volume of 85 cm^3^ according to the recipes shown in Table 1 and the formulation shown in Table 2. The Applied compounding parameters are described in Figure 2.

The compound-filling factor was set at 0.75, corresponding to a volume of 63.75 cm^3^. The temperature of the mixer was set and maintained at 80 °C for the NP1 and NP2 stages and 60 °C for the PR stage. Between mixing stages, the compounds were further homogenized by being passed through a two-roll mill 6 times with a roller rotation speed of 32 rpm, then through another roller at a speed of 24 rpm with a gap of 2 mm. A total of 8 batches of compounding were performed with different amounts of silica, chemically modified MFCs, and TDAE processing oil (shown in Table 2). An amount of 50 g of the processed green compounds was vulcanized under hot pressing at 150 °C and 170 bar for 30 min.

### 2.4. Characterizations

#### 2.4.1. Chemical Composition Analysis

Chemically modified MFCs were ground with a microfine grinder (IKA, MF 10.1; IKA^®^-Werke GmbH, Breisgau, Germany) through a 1 mm screen. Ground samples were hydrolyzed using a two-step sulfuric acid process [19]. After acid hydrolysis, the hydrolysate was collected for monosaccharide analysis, and Klason lignin content (acid-insoluble residues) was gravimetrically determined. The chemical composition of the hydrolysate was analyzed by high-performance anion-exchange chromatography with pulsed amperometric detection (HPAEC-PAD) [20].

#### 2.4.2. Attenuated Total Reflectance–Fourier Transform Infrared Spectroscopy (ATR-FTIR)

ATR-FTIR analyses of unmodified and chemically modified MFCs were carried out using a Thermo Scientific Nicolet iS50 FT-IR spectrometer. For the analysis, unmodified and chemically modified MFC films were applied to a diamond cell, and the transmission spectra between 400 and 4000 cm^−1^ were measured at room temperature.

#### 2.4.3. X-Ray Diffraction (XRD)

X-ray diffraction was performed on unmodified and modified MFCs using a Bruker AXS X-ray diffractometer equipped with a filtered Cu Kα radiation source (λ = 0.1542 nm) at an operating voltage and current of 45 kV and 40 mA, respectively, using a 2D detector. The sample crystallinity index (CI, %) was calculated from the XRD spectra using the Segal method based the height of the 200 peak (*I*_200_, 2θ = 22.7°) and amorphous peak at 2θ = 18° (*I_AM_*) between the 200 and 110 peaks (Equation (1)) [21]. *I*_200_ represents the sum of crystalline and amorphous material, while *I_AM_* represents amorphous material only.

(1) $$CI\left(\%\right)=\frac{I_{200}-I_{AM}}{I_{200}}$$

#### 2.4.4. Morphological Characterization

Unmodified and modified MFCs were first deposited on a STEM grid of a carbon film deposited on 400 mesh Cu. The morphology of MFCs was subsequently observed using a focused ion beam (FIB) scanning transmission electron microscope (STEM) under a transmission mode operated at 30 kV.

#### 2.4.5. Thermogravimetric Analysis (TGA)

Dynamic thermogravimetric measurements were performed using a Discovery TGA TA instrument (New Castle, DE, USA). Temperature programs for dynamic tests were run from room temperature to 700 °C at a heating rate of 10 °C/min. The tests were carried out under a nitrogen atmosphere (25 mL/min) and in an air atmosphere (25 mL/min).

#### 2.4.6. Moving Die Rheometer (MDR)

The curing behavior of the prepared compounds was evaluated by MDR with an MDR 2000 rheometer (Alpha Technologies, Bellingham, WA, USA) at a frequency of 1.667 Hz, a strain of 0.5 degrees, and a temperature of 160 °C for 60 min. Samples were 43 mm in diameter and 2 mm thick.

#### 2.4.7. Dynamic Mechanical Analysis (DMA)

Dynamic mechanical testing was carried out on a GABO Eplexor DMA. The temperature dependence of the viscoelastic properties were measured from −80 °C to 80 °C at a frequency of 10 Hz, dynamic strain amplitude of 0.5%, and static strain of 1%. The temperature was increased in steps of 1 °C, and the sample was thermally equilibrated before testing at each temperature. A 150 N load cell was used to perform measurements on cured rubber with a rectangular specimen geometry of 6.35 mm × 37 mm × 2 mm. The phase angle (δ) and E*, which is the magnitude of the complex modulus, were directly determined by testing. Then, the storage modulus (*E*′), loss modulus (*E*″), and tan delta (tanδ) were calculated according to the following formula.

*E*′ = E* × cos*δ*; *E*″ = E*× sin*δ*; and tan*δ* = *E*″/*E*′ (2)

#### 2.4.8. Tensile Test

Tensile specimens were cut from the 2 mm thick cured rubber sheets in the mill direction using a DIN 53504-S2 (22) cutting die with a gauge length of 50 mm and a width of 4 mm. The tensile testing of each specimen was performed using an Instron Model 5864 Electro-Mechanical Test Instrument with a 1 kN load cell (Instron Corp., 2525–806 1 kN; Norwood, MA, USA). Each specimen was extended at a crosshead rate of 200 mm/min until the break. The tensile measurements were conducted by testing 3 specimens for each sample under ambient conditions; standard deviations of the results are presented in the relevant section.

## 3. Results and Discussion

### 3.1. ATR-FTIR Analysis

**Figure 3:**
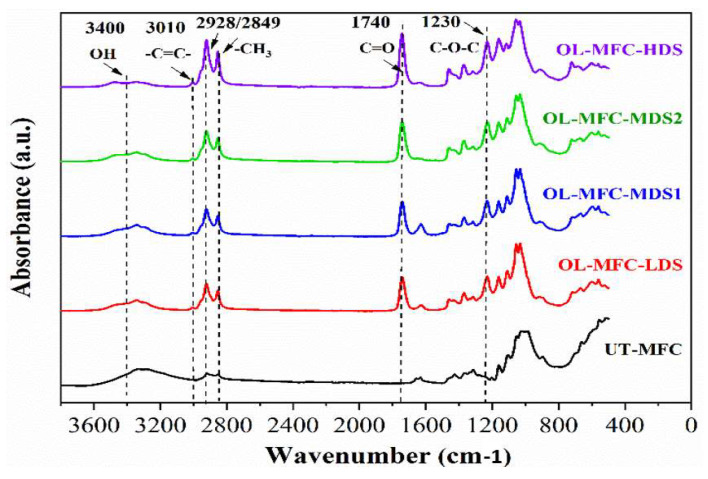
FTIR spectra of untreated MFC and chemically modified MFC with different degrees of substitution (DS).

Oleic modified MFC (OL-MFC) was synthesized by reacting oleoyl chloride on the hydroxyl groups of MFCs in pyridine (Figure 1). An excess amount of oleoyl chloride was applied to achieve OL-MFCs with various degrees of substitution (DS = 0.24–0.91). The success of the esterification modification of MFCs was confirmed by FTIR spectroscopy (Figure 3). Compared with the FTIR spectra of unmodified MFCs, the characteristics of grafted ester pendant groups indicated by a carbonyl C=O stretching vibration at 1740 cm^−1^, antisymmetric C-O-C stretching at 1230 cm^−1^ [22], and alkenyl C=C stretching at 3010 cm^−1^ were observed for all modified MFCs, confirming the successful modification of MFCs with oleoyl chloride. With the increase in DS, an increase in the intensity of the carbonyl C=O peaks, the peaks at 3010 cm^−1^ assigned to C=C stretching, and the peaks at 2928 and 2849 cm^−1^ assigned to C-H stretching vibrations was observed. The increased intensities confirmed the more pendant groups of oleoyl chloride were grafted onto the MFC backbone. Furthermore, the low intensity of O-H at 3400 cm^−1^ demonstrates that large OH groups on the modified MFCs were replaced with the hydrophobic aliphatic chain of oleoyl chloride.

### 3.2. XRD Analysis

**Figure 4:**
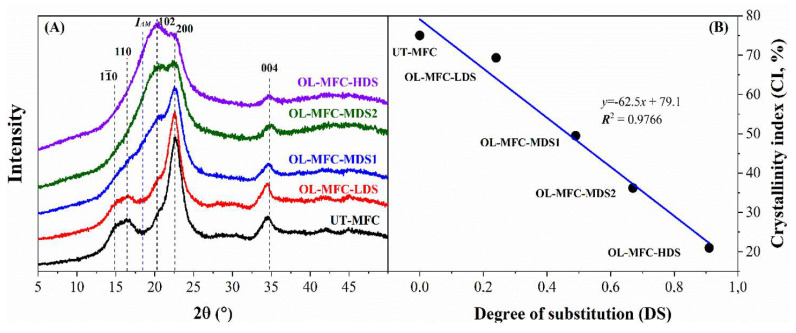
XRD spectra of unmodified MFCs and chemically modified MFCs with different degrees of substitutions (**A**) and crystalline index (CI) values vs. degree of substitutions (**B**).

The unmodified MFCs exhibited very high cellulose content of up to 95% as indicated by glucan content (Table 3) and low hemicellulose content of about 3% (indicated by xylan and mannan) and low Klason lignin of 0.3%. As a result, a high crystallinity index (CI) of up to 75% for the cellulose-rich MFCs was measured (Figure 4), which is consistent with what has been reported for unbleached pulps and MFCs produced from unbleached pulps [23,24,25]. The XRD patterns shown in Figure 4 indicate the transformation of the cellulose crystal structure with an increase in DS after chemical modification. As can be seen from the spectra, the unmodified and OL-MFC-LDS samples exhibit a similar and typical cellulose Iβ crystalline structure with characteristic peaks at 2θ = 14.9°, 16.7°, 20.6°, 22.7°, and 34.4° for the 1$\bar{1}$0, 110, 021, 200, and 004 diffraction planes, respectively [21,26].

With a further increase in DS, two diffraction planes (1$\bar{1}$0 and 110) almost disappeared in the XRD spectra of the modified MFCs, and the intensity of the peaks corresponding to the 200 and 004 diffraction planes became weak (Figure 4). In contrast, the peak at 2θ = 20.6° corresponding to the 200 diffraction plane increased with increased DS values, becoming the most intensive peak in the XRD patterns of OL-MFC-HDS.

In addition, the intensity of the peak at 2θ = 18°, which was attributed to the contribution of amorphous components of the materials, increased consistently in parallel with that of the peak at 2θ = 20.6°. These changes indicate that the crystalline structure of cellulose was greatly altered when DS was above 0.24. The alteration of the crystalline properties of MFCs due to high grafting should be avoided, as crystallinity contributes to the mechanical properties of MFCs and MFC composites. Similar results have been observed for both heterogeneously [27] and homogeneously [26] modified cellulose fibers. However, the changes in the intensity at 2θ = 14.9° and 16.7° are not consistent with the study reported by Almasi et al. (2015), where the intensity of the two peaks was found to remain constant during the esterification of freeze-dried MFCs with oleic acid at comparable DS values [18]. This is presumably due to the differences in the conditions applied for the modification.

The unmodified MFCs had the highest CI of up to 75%, while a progressive decrease in the CI alongside an increase in the DS values of the chemically modified MFCs was observed. The CI of OL-MFC-LDS was 69%, decreasing to 49% for OL-MFC-MDS1, 36% for OL-MFC-MDS2, and 21% for OL-MFC-HDS (Figure 4). Interestingly, the CI showed a negative linear correlation with the DS of modified MFCs, with a slope of −62.5. This further confirms the damage of the cellulose crystallinity due to the introduction of fatty acid hydrocarbon chains into the cellulose polycrystalline domains.

### 3.3. STEM Morphology

**Figure 5:**
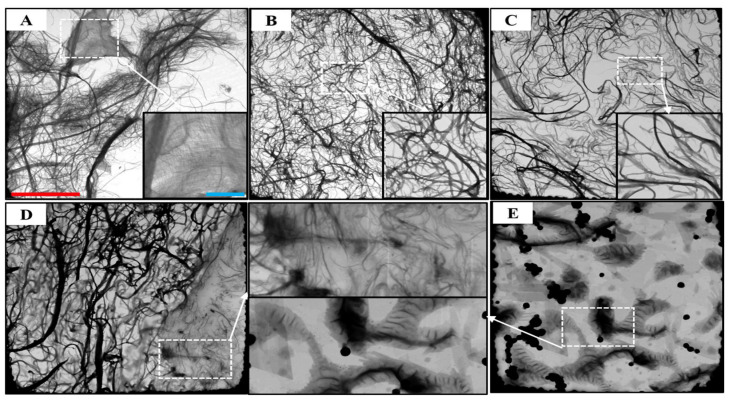
STEM images of untreated MFC and chemically modified MFC with different degrees of substitution ((**A**)—UT-MFC; (**B**)—OL-MFC-LDS; (**C**)—OL-MFC-MDS1; (**D**)—OL-MFC-MDS2; (**E**)—OL-MFC-HDS) (red scale bar in image A represents 10 µm, and images (**B**–**E**) have the same scales; the blue scale bar in image A represents 2 µm, and all magnified images have the same scale).

Chemical modification of MFCs starts with the easily accessible OH groups. Subsequently, it proceeds to the amorphous regions of the cellulose at the initial stage, even with a low dosage of modifier (e.g., OL-MFC-LDS and OL-MFC-MDS1). This results in modified MFCs with improved swelling capacity in the solvents, alongside a preserved three-dimensional network morphology (images B and C in Figure 5). Large nanofibrils were observed on the modified MFCs due to aggregations upon drying (images B and C vs. image A in Figure 5). The increased swelling capacity of the MFCs in the solvent allows for the diffusion of reagents and modifiers deep into the amorphous spaces of the fibrils, followed by esterification with the internally available hydroxyl groups. This contributes to the disruption of hydrogen bonding between fibrils or cellulose chains [28]. Consequently, individual fibrils could be separated from the bundles of fibrils, and cellulose chains could probably be detached from the surface fibrils with extended modification under a high dosage of chemical reagents (i.e., OL-MFC-MDS2) (image D in Figure 5).

When the degrees of substitution increased for OL-MFC-MDS2, the disruption in the OH interaction could not destroy the network structure of the nanofibrils. With a further increase in the DS value to 0.91 (i.e., OL-MFC-HDS), the network structure and the micro-fibrillar integrity were entirely lost (image E in Figure 5). There is the possibility that the disruption of the hydrogen bond network of MFCs at high grafting could degrade the fibrils and engineer a new type of interaction. A possible Van der Waals type of interaction could convolute the fibrillar morphology into a new structure, as shown in Figure 5E. At higher magnification, it appears that the agglomerates are most likely comprised of the degraded and aggregated fiber and fibril fragments. Degradation of MFCs due to surface modification was previously reported [29]. A series of chemically modified MFCs with varied DS values were reported, with convincing evidence that showed a clear evolution in the morphology of MFCs with extended surface modification. Overall, the evolution of the morphology of MFCs shown in Figure 5 shows the progressive degradation of nanofibrils and cellulose chains because of the continuous introduction of fatty acid side chains into both amorphous and polycrystalline domains of cellulose. These results are consistent with the changes in the XRD spectra patterns shown in Figure 4.

### 3.4. Thermal Stability Analysis

**Figure 6:**
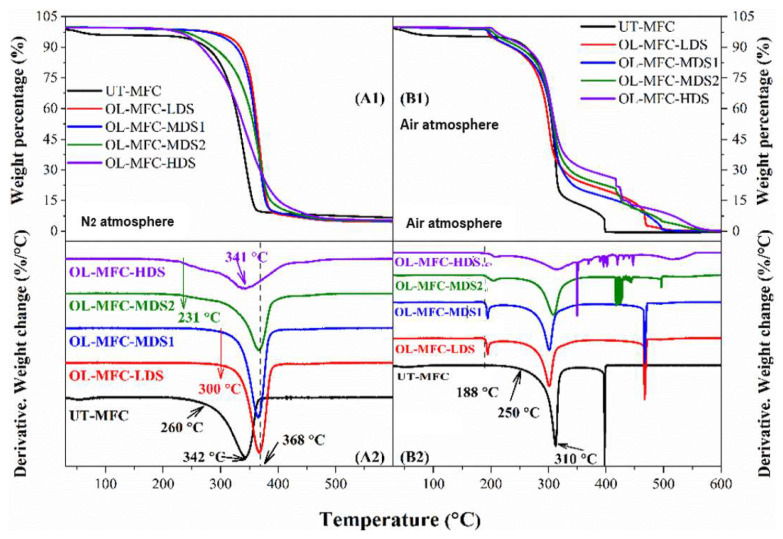
Thermogravimetric analysis (TGA) of unmodified MFCs and chemically modified MFCs in a N_2_ atmosphere (**A1**,**A2**) and air atmosphere (**B1**,**B2**). (**A1**,**B1**) Thermogravimetric (TG) curves; (**A2**,**B2**) derivative thermogravimetric (DTG) curves.

Studies on the thermal stability of the chemically modified MFCs under nitrogen and air atmospheres are presented in Figure 6. The unmodified MFCs featured a substantial decomposition around 260–380 °C, representing the degradation of the cellulose backbone, with a minor weight loss below 150 °C due to the loss of volatiles and moisture [26,30,31]. The MFCs comprise up to 95% cellulose, with little hemicellulose and lignin (Table 3) and negligible weight loss at temperatures ranging from 150 to 260 °C and above 380 °C [32,33,34] under nitrogen and air atmospheres.

Upon chemical modification, distinct changes in the thermogravimetric analysis (TGA) and the derivative thermogravimetric (DTG) curves were observed when compared to the unmodified MFCs. Compared to the unmodified MFCs, the weight loss remained stable below 150 °C, irrespective of the test condition (i.e., air or N_2_), which may be a result of the hydrophobic character of the grafted oleoyl groups. A similar finding was reported for cellulose laurate esters [26]. In a N_2_ atmosphere (graphs A1 and A2 in Figure 6), similar degradation behavior above 150 °C was observed for all the chemically modified MFCs compared with that of the unmodified MFCs.

At low DS values up to 0.49, the onset decomposition temperature shifted to a higher temperature from about 260 °C to 300 °C, implying an increase in thermal stability after the esterification reaction. This improvement in thermal stability suggests a rearrangement on the cellulose backbone to form a new ordered structure because of the long-chain fatty acid groups [26,35]. However, at very high grafting, the impact on the thermal stability became pronounced. The high level of grafting possibly led to the degradation of the fibers (STEM images, Figure 5) because of the large disruption of the MFC microstructure. This likely exposed the cellulose structure to easy thermal degradation. Although the polycrystalline structure of cellulose was partially altered or damaged, the results are in line with previous studies [26,35,36,37].

On the contrary, the samples with higher DS values (i.e., 0.67 and 0.91) started to decompose at a lower temperature of about 230 °C, which is 30 °C lower than that of the unmodified MFCs. The morphological changes—mainly the rearrangement of cellulose chains and their counterparts, as seen in Figure 5—are more likely to be responsible for the decreased onset decomposition temperature. Different TGA and DTG patterns were recorded in the air atmosphere due to the oxidation reactions of the attached fatty acid groups. All the highly modified MFCs remained stable up to 188 °C, which is about 70 °C lower compared to the 260 °C for the unmodified MFCs, and began to decompose thereafter. The decrease in the initial decomposition temperature is presumably due to the low stability of double bonds in the attached fatty acid groups [38]. Before the onset of decomposition, a slight gain of mass was observed because of the uptake of oxygen at the beginning of the oxidation of the unsaturated bonds [38,39]. The decomposition rate was almost constant from 200 to 260 °C and became substantially higher from 260 to 350 °C owing to the progressive oxidation of the remaining alkyl chains attached to the cellulose. The last decomposition stage was recorded between 350 and 580 °C, where the total oxidation of the carbonaceous residues formed in the former stage occurred [38] in a dynamic oxidation atmosphere for all samples.

### 3.5. Compounding and Rubber Compound Properties

#### 3.5.1. Processability Analysis

**Figure 7:**
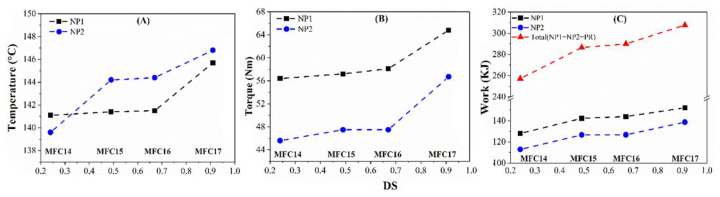
The changes in the maximum mixing temperature (**A**), the maximum rotor torque (**B**), and work done (**C**) in different mixing stages with increased DS of chemically modified MFCs (from MFC14 to MFC15, MFC16, and MFC17).

The processability of modified MFCs was studied from the maximum temperature (T_m_), maximum rotor torque (T_qm_), and work done during each stage of compounding (Table 4). The degree of substitution and the formulations of the modified MFCs had a considerable influence on the processability of the modified MFCs. Generally, the maximum temperature, the maximum rotor torque, and the work done in all the mixing stages (NP1, NP2, and PR) increased relative to the increase in the DS. The T_m_ and T_qm_ in NP2 and the total work done for compounding of the MFC14 sample with OL-MFC-LDS were 139.6 °C, 45.6 Nm, and 257.2 KJ, respectively. With an increase in DS for OL-MFC-MDS2 (MFC16), the values increased to 144.4 °C, 47.5 Nm, and 289.9 KJ, respectively, corresponding to 3%, 4%, and 13% compared to MFC14. As the DS further increased in sample MFC17, those values increased by 5%, 24%, and 20%, respectively, compared to MFC14 (Figure 7).

The compounding process demonstrates the exothermic heat of mixing involving strong interactions between molecules or nano- or micro-sized particles, probably along with chemical reactions. The increase in the compounding parameters alongside the increase in the DS values of MFCs attests to the improved interactions between the modified MFCs and other materials in the mix. The increases in the mixing temperature, rotor torque, and work done with increased DS values from MFC14 to MFC15 (Figure 7) are primarily due to the enhanced surface hydrophobicity of MFCs, leading to improved elastomers/modified MFCs and modified MFC/silanized silica interactions. With further increases in the DS for MDS2, the increase in those compounding parameters plateaued (Figure 7), showing that the hydrophobicity of MFCs reached the highest achievable level without losing the fibrillar network (Figure 5). However, another sharp increase was observed in those compounding parameters from MFC16 to MFC17 (HDS). This is assumed to be related to both the enhanced hydrophobicity of MFCs and the significant alteration of the morphologies. As shown in Figure 5, the supposed delamination of the surface fibers of cellulose chains and the rearrangement of the cellulose microstructure occurred due to the enhanced hydrophobicity.

**Figure 8:**
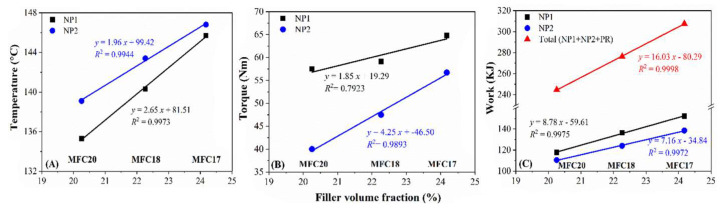
Changes in the maximum mixing temperature (**A**), maximum rotor torque (**B**), and work done during compounding (**C**) with increased total filler volume fraction (from compound MFC17, to MFC18 and MFC20).

Furthermore, impacts of the silica dosage and the total amount of processing oil on the compounding parameters were noticed (Table 4 and Figure 8). With the removal of 10 phr silica, decreases in the maximum mixing temperature, the maximum rotor torque, and work done in the mixing stage of NP1 and NP2 were observed due to the decreasing viscosity of the mixtures occasioned by the low filler volume, which could also explain the drop in the mixing temperature [40].

#### 3.5.2. MDR Analysis

**Figure 9:**
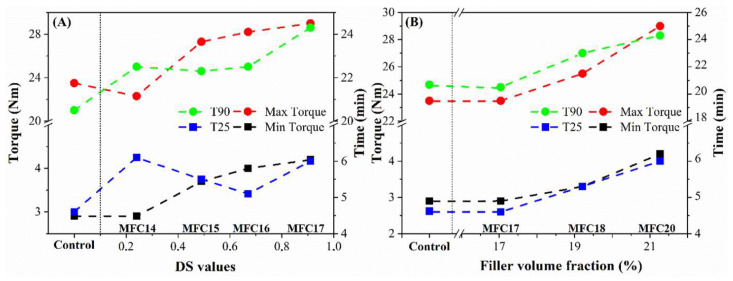
Changes in the maximum torque, minimum torque, T_25_, and T_90_ with increased DS values of the modified MFCs (**A**) and with increased filler volume fraction (**B**). (*T_25_, time required for 25% cure development; T_90_, time required for 90% cure development*).

The cure characteristics of the compounds were studied using a moving die rheometer (MDR), as presented in Table 5 and Figure 9. The maximum torque (T_max_) represents the achieved crosslink density and the degree of reinforcement of the filler in the matrix. The minimum torque (T_min_) alludes to the viscosity of the green compounds and the interactions between the fillers and the matrix. It can be observed that the DS of the modified MFCs impacted the maximum and minimum torque, which increased with increasing DS and filler volume fraction. This can be attributed to the possible contributions of the double bonds of the aliphatic chains to the vulcanization reaction. Thereafter, the T_min_ and T_max_ torque plateaued when DS values were above 0.67 (i.e., OL-MFC-MDS2). The progressive increase in the maximum and minimum torque with the increase in filler volume fraction is simply due to the increased crosslink contributions of the increase in filler volume.

The changes in the cure kinetics at 25% cure (T_25_) and optimum cure time (T_90_) were observed to be partly influenced by the DS and filler volume fraction, as compounds with modified MFC showed a distinct behavior relative to the control. The t_25_ maintained a duration of 4.6–6.1 min relative to the control, regardless of the degree of substitution. It was also observed that the optimum cure time of the compounds with modified MFCs reached 24.3 min compared with the control (20.5 min). This can likely be attributed to the low reactivity of the fatty acid modifier with other crosslinking agents (e.g., TESPT silane and sulfur), especially when a high dosage of the modifier was incorporated (i.e., for high DS of MFCs). The contributions from the aliphatic double bonds to the crosslinking may have also further delayed the curing time, requiring more time to utilize the double bond in the crosslinking process. Furthermore, the progressive increase in t_25_ and t_90_ as filler loading increased is presumably due to the increased in surface area because of the high filler loadings [40,41]. Overall, the highest optimum cure t_90_ was achieved at about 24 min. Therefore, it would be advantageous to maintain a cure window not exceeding 30 min at the same curing temperature (i.e., 150 °C). This would result in good compound performance and avoid overcuring with possible reversions.

#### 3.5.3. Rubber Process Analysis (RPA) and Dynamic Mechanical Analysis (DMA)

**Figure 10:**
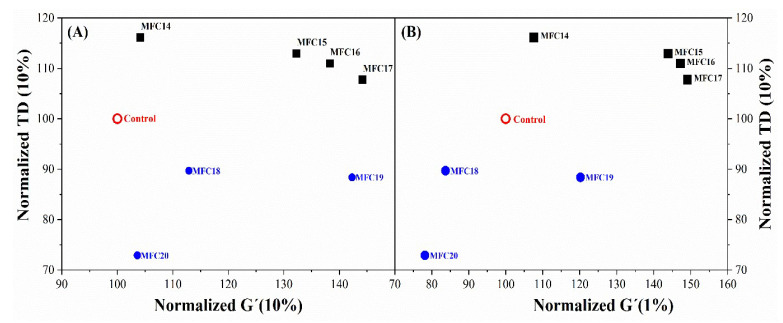
Normalized TD (10%) versus G′ (10%) (**A**) and G′ (1%) (**B**) for silica/chemically modified MFC hybrid rubber composites measured using an RPA.

The dynamic mechanical behavior of the fabricated MFC/silica/SBR rubber compounds was studied and compared with that of a silica/SBR compound as a control. Figure 10 shows the normalized tangent delta (TD) alongside the storage modulus (G′) at 1% and 10% strain. An ideal compound is required to have an acceptably high level of stiffness (G′) and low tangent delta (TD). These properties change during strain-imposed deformation because of the filler network breakdown. It can be observed that compounds with higher stiffness (G′) tend to have a higher TD and vice-versa. The tradeoff between stiffness and damping at 1% strain presented MFC19 as a compound with interesting behavior. A further increase in the strain to 10%, indicated MFC18 and MFC19 as having a better tradeoff compared to the control. These changes along the progressive low strain demonstrate the extent of filler network breakdown and recovery described as the Payne effect [42,43]. At low strain up to 10%, good stiffness and TD were retained for MFC18 and 19 compared to the control. This is an indication that the filler network of these compounds was resilient and was not subject to considerable damage. A low Payne effect such as that seen in these compounds could be useful for tire tread applications because the tread is subjected to dynamic deformations and requires compounds with low energy dissipation resulting from filler network breakdown. These properties were achieved by modulating the DS and compound recipe for a better tradeoff. The two compounds were made with 20 phr less silica compared to the reference. Eventually, a reasonable level of stiffness was maintained while keeping an acceptable level of TD. The stiffness could be further enhanced at low filler loading by reducing the TDAE oil. These results demonstrate that a percolation threshold needed for reinforcement can be achieve at low filler loading by substituting 10 phr of modified MFC with 20 phr of silica.

The high level of stiffness observed in some compounds (MFC15, 16, and 17) offers new opportunities for applications of MFC-reinforced polymer in aspects of tires not requiring high damping properties.

The behavior of the compounds was further studied at different temperatures. Some of the properties studied include the tangent delta at 60 °C, which is used as a rolling resistance indicator [44], and the tan delta at 0 °C, which is used as an indicator for wet traction [45], as well as the modulus of the compounds at 30 °C. The influences of DS of MFCs and filler loadings on the modulus, rolling resistance, and wet traction of the compound are shown in Table 6. Above a DS of 0.67 (MFC16), a decrease in the modulus was observed. This reduction in the modulus could possibly be attributed to the loss of the reinforcing fibrillar properties of the MFC due to grafting. A progressive loss of crystallinity and compromised morphology were observed. These crucial fiber properties are important determinants of the properties of the final compound, especially the modulus. Given the comparable values of the tan delta of the modified MFC and control, an appreciable decrease in T_g_ of the compounds made with MFCs provides greater flexibility for applications. The low T_g_ values of the MFC compounds are probably due to the low T_g_ of the fatty acid modifier compared to that of the TDAE processing oil [46].

The improved modulus compared to the control also confirms the high reinforcing efficiency of the MFCs in the elastomeric matrix. Alongside the appropriate degree of substitution (not compromising the fiber properties), the modulus can be enhanced by good interfacial adhesion [47,48].

#### 3.5.4. Tensile Properties

**Figure 11:**
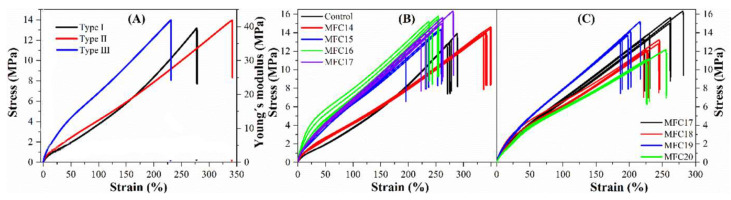
Tensile curves of (**A**) dominant tensile behavior of MFC/silica/SBR and silica/SBR compounds; (**B**) tensile evolution of the control, MFC14, 15, 16, and 17; and (**C**) tensile curves of MFC17, 18, 19, and 20.

The stress–strain behaviour of all investigated samples is shown in Figure 11 and tensile properties shown in Table 7. Three typical tensile behaviors were observed (graph A in Figure 11). The first behavior (type I) of the referenced silica-filled SBR/PBD compound is characterized by strain-dependent modulus indicated by an upturned curve, leading to increased slope as the strain increases, which could be due to the limited chain extensibility [43]. The second behavior (type II) is linearly elastic, corresponding to a straight stress–strain curve. The last behavior (type III) includes some plastic flow, as indicated by a slightly bent curve at low deformations (1–50%), followed by linearly elastic behavior until failure.

The compound with the lowest DS (i.e., MFC14) exhibited type II behavior. However, an increase in the DS of MFCs resulted to compounds showing some plastic deformation at low strains (1–50%, type III behavior). The plastic deformation at low strains became the most pronounced for the compounds incorporated with MFC16. This could be due to the evolution of plasticized domains on the fibers from the hydrophobization. Ordinarily, the weight of the fatty acid chain can promote delamination of the surface fibers and make them function as plasticizers instead as reinforcing agents. This, in turn, could lead to the slippage of the hydrophobic cellulose chains on the surface against the relative hydrophilic internal cellulose chains towards the direction of the external load [48]. As the DS value further increased to 0.91, the resulting compounds (MFC17, 18, 19, and 20) exhibited decreased plastic deformation. This could be a result of the reduced sizes and rearrangement of cellulose chains (Figure 5), with enhanced interactions keeping the chains from sliding towards each other.

Apart from the changes in the stress–strain behavior, a significant increase in Young’s modulus of the compounds was observed at low strains (<50%) with increased DS (graph B in Figure 11). The highest values of the Youngs’s modulus at 25% was determined to be 8.1 MPa for the MFC16 compounds, which is 1.8 times higher than that of the referenced silica-filled compounds. There was also a rise in the modulus at higher strains increased DS. For example, Young’s modulus at 100% strain increased gradually with increased DS values and reached about 5.0 MPa when the DS was above 0.49 (i.e., MFC16). This in line with previous observations [49,50,51,52,53,54], further confirming the high reinforcing efficiency of MFCs in rubber composites. In addition, a high tensile strength of 15 MPa was achieved with the addition of modified cellulose fibers compared with the reference compound made of silica (13 MPa). The strain at break was higher for the MFC14 compound, while it started to decrease as the DS further increased, resulting in more brittle compounds. The results, in general, are consistent with previously reported findings that the incorporation of cellulose fibers can result in a significant improvement in the modulus and, to a lesser extent, in the tensile strength.

## 4. Conclusions

The chemical modification of MFCs with oleoyl chloride provided hydrophobic domains needed for good dispersion in an SBR matrix and good filler/polymer interactions. MFCs with a DS of 0.2–0.9 were successfully synthesized in pyridine and compounded with silica and SBR. The compounds were fabricated by substituting 10–30 phr silica with 10 phr of modified MFCs. As the DS values increased, significant impacts on the morphology and crystallinity of modified MFCs were observed. These changes were found to further affect the processability of the compounds, as well as the curing behavior, modulus-rolling resistance tradeoff, and tensile properties of the resulting rubber compounds. Overall, the highest reinforcement was achieved for the MFC compound with a DS value of 0.67 (i.e., MFC16). This compound had a high surface hydrophobicity and also retained the fibrillar network structure. The most important drawback in the compound properties was the wear abrasion properties (results not presented), which require improvement. However, this study demonstrates that a small amount of MFCs can be used to replace a large amount of silica, resulting in improvements in reinforcement and mechanical properties. The improved properties achieved with the incorporation of modified MFCs opens potential applications for the use of sustainable bio-based materials to produce tire compounds and lightweight composites.

## Figures and Tables

**Table 1:** The general composition of the rubber compounds.

Stage	Composition	Amount (phr)
**NP1**	Polystyrene butadiene	80
	Polybutadiene	20
	TDAE oil	3.75–25
	Zinc oxide	0.5
	Stearic acid	3
	Silica	50–80
	MFCs	10
**NP2**	6PPD	2.5
	TESPD silane	8
	Silica	15
**PR**	Zinc oxide	2
	Sulfur	1.1
	MBT	0.3
	DPG	3.2
	CBS	2.3

**Table 2:** Rubber compounds and their compositions.

	Type of MFC	Degree of Substitution (DS)	Amount (phr)		
			Silica	MFCs	TDAE
Control	/	/	80	0	25
MFC14	LDS	0.24	70	10	21
MFC15	MDS1	0.49	70	10	17
MFC16	MDS2	0.67	70	10	14
MFC17	HDS	0.91	70	10	10
MFC18	HDS	0.91	60	10	10
MFC19	HDS	0.91	60	10	3.75
MFC20	HDS	0.91	50	10	10

**Table 3:** The chemical compositions of unmodified MFCs used in this study.

Amount (%)					
Arabinan	Galactan	Glucan	Xylan	Mannan	Klason Lignin
n.d.	n.d.	94.5 (2.8)	2.1 (0.2)	1.2 (0.1)	0.3 (0.2)

**Table 4:** A summary of compounding parameters during each mixing stage for all rubber compounds.

Sample	NP1		NP2		PR		Total Work W (KJ)
	Tm (°C)	W (KJ)	Tm (°C)	W (KJ)	Tm (°C)	W (KJ)	
**Control**	147.9	133.8	138.1	117.5	77.6	14.6	265.9
**MFC14**	141.1	128	139.6	112.9	79	16.3	257.2
**MFC15**	141.4	142.2	144.2	126.6	80.8	17.7	286.5
**MFC16**	141.5	143.9	144.4	126.7	80.5	19.3	289.9
**MFC17**	145.7	152.2	146.8	138.6	81.7	16.8	307.6
**MFC18**	140.3	136.5	143.4	124	77.4	15.9	276.4
**MFC19**	143	152.5	147.7	141.7	83.6	19.8	314
**MFC20**	135.3	117.8	139.1	110.5	78.1	16.4	244.7

**Table 5:** Vulcanization characteristics of each rubber compound measured by a moving die rheometer (MDR).

Sample	Min Torque (T_min_) (Nm)	Max Torque (T_max_) (Nm)	25% Cure T_25_ (min)	Optimum Cure T_90_ (min)
Control	2.9	23.5	4.6	20.5
MFC14	2.9	22.3	6.1	22.5
MFC15	3.7	27.3	5.5	22.3
MFC16	4.0	28.2	5.1	22.5
MFC17	4.2	29.0	6.0	24.3
MFC18	3.3	25.5	5.3	23.0
MFC19	4.5	29.6	4.7	22.0
MFC20	2.9	23.5	4.6	20.5

**Table 6:** Dynamic mechanical analysis (DMA) results for the referenced rubber compounds and the rubber compounds with chemically modified MFC fibers.

Sample	T_g_ (°C)	TD (0 °C)	TD (60 °C)	E′ (MPa, 30 °C)
Control	−21.8	0.306	0.081	12.6
MFC14	−24.0	0.258	0.095	20.5
MFC15	−24.2	0.228	0.107	36.2
MFC16	−24.8	0.205	0.111	56.3
MFC17	−23.6	0.241	0.097	29.7
MFC18	−23.1	0.249	0.084	21.5
MFC19	−23.1	0.236	0.087	28.8
MFC20	−23.3	0.237	0.080	17.6

**Table 7:** Tensile properties of rubber compounds with chemically modified MFC fibers. Values are shown as mean (standard deviation).

Sample	Tensile Strength (MPa)	Young’s Modulus (MPa)	Strain (%)
Control	13.1 (0.5)	3.8 (0.0)	277.1 (6.9)
MFC14	14.2 (0.4)	3.4 (0.1)	343.2 (5.7)
MFC15	13.4 (1.2)	4.7 (0.3)	245.2 (13.3)
MFC16	15.0 (0.7)	4.7 (0.1)	243.9 (10.0)
MFC17	14.8 (1.2)	5.1 (0.1)	251.8 (24.4)
MFC18	12.4 (0.6)	4.2 (0.1)	233.3 (11.0)
MFC19	13.8 (0.8)	5.8 (0.1)	198.5 (12.0)
MFC20	11.5 (0.6)	3.7 (0.1)	238.4 (15.6)

## Data Availability

Data is contained within the article.
